# Behavior of mandibular canines as abutment teeth and indirect retainers in Kennedy class II Removable Partial Denture Prosthesis

**DOI:** 10.1016/j.heliyon.2018.e00575

**Published:** 2018-03-15

**Authors:** Marisol C. Camacho, Yolanda R. Gallardo, Roberto Ch. Stegun, Bruno Costa, Newton Sesma

**Affiliations:** a Department of Prosthodontics, Dental School, Científica del Sur, Peru; b Department of Prosthodontics, Dental School, Sao Paulo University, Av. Prof. Lineu Prestes, 2227- Cidade Universitária- Butanta, São Paulo, CEP: 05508-000, Brazil

**Keywords:** Materials science, Dentistry, Engineering

## Abstract

**Purpose:**

The purpose of this study was to evaluate the behavior of mandibular canines acting as abutment teeth and indirect retainers of a Kennedy class II according to different designs: lingual rest and lingual rest associated with a reciprocal arm.

**Materials & methods:**

A resin cast with two simulated canine teeth was made in Ni-Cr alloy, representing a Kennedy class II mandibular arch. With the objective of simulating the resilience of the periodontal ligament, a polyurethane layer was added at the canine tooth's root. A metallic framework of Co-Cr alloy was fabricated with a T bar clasp and a lingual rest associated with a reciprocal arm. To obtain the second framework, the reciprocal arm was removed using a tungsten bur. Each framework was submitted to tensile force using a VersaTest machine. The magnitude and direction of canine movement during removal of the framework was measured using two dial gauges (mm). The axial tensile force required to remove the experimental framework (N) was also evaluated. The data were compared using the paired t-test with 95% confidence intervals. Differences were considered significant at P < .05.

**Results:**

The mean retentive force of the modified design framework with the reciprocal arm was significantly higher (P < .0001) than that of the framework with the lingual rest. The abutment teeth showed movement in the lingual and mesial directions, and this movement was less when associated with the reciprocal arm design.

**Conclusion:**

The reciprocal arm in association with a lingual rest in the framework decreased the movement of the abutment teeth when analyzed in the bucco-lingual and mesio-distal directions and contributed to increased retention by friction.

## 1 Introduction

Within the various treatment options available for replacing teeth in partially edentulous patients, treatment with a Removable Partial Denture Prosthesis (RPDP) is still used because it is an effective, conservative, and affordable option that provides adequate plaque control and requires frequent maintenance visits [1, 2, 3, 4, 5]. It is fundamentally important to carefully design the RPDP, taking into account all the biomechanical principles needed to achieve a homogeneous distribution of occlusal forces and a regular adaptation of the oral tissues. Unfortunately the RPDP, has been relegated to a second term in favor of more expensive treatments but of greater functionality and aesthetics, as are the fixed prosthesis on natural teeth or on implants, reason why there is increasingly less scientific evidence on this issue in the past 20 years [6, 7, 8].

Frequently, distal extension removable partial dentures have been implicated in increased mobility and destruction of the supporting tissues of abutment teeth [3, 9, 10]. Differences in resilience between periodontal abutment tissue and residual ridge mucosa are generally recognized as the main problem with this type of treatment [4, 7, 11, 12]. Moreover, the amount of stress transferred to the abutment tooth depends on the rest location, clasp design, connector rigidity, direction and magnitude of the force, denture base extension, and angulation of the residual ridge [4, 9, 10, 13, 14, 15, 16, 17, 18].

Another important component of an RPDP is the indirect retainers; they prevent the retentive clasp arms from becoming a fulcrum from which the denture will rotate when the bases move away from the residual ridge [19, 20].

In a Kennedy class II RPDP design, it is recommended that the bar clasps be placed on the edentulous side because this provides a more favorable force distribution, less contact with the abutment teeth, better aesthetics and less interference with the natural teeth contours [6, 7, 8, 9, 17].

Due to the anatomy of lingual and/or palate faces, it is very difficult for the canine teeth to provide reciprocity with only a lingual rest. The placement of another component in the RPDP design satisfies this condition of reciprocity, for example, in surveyed crowns [21, 22, 23, 24]. Reciprocity is effective when the force applied to the teeth by the retention arm is balanced by the rigid element of the framework during removal and insertion of the RPDP. This prevents the abutment from being exposed to deleterious horizontal forces, as well as increases clasp retention [25, 26].

In the case of a canine tooth, the first element of the RPDP to move during gingival-occlusal movement is the rest, losing the principle of reciprocity. The purpose of this study was to investigate the behavior of the mandibular left canine, acting as an abutment in a Kennedy class II RPDP, according to different designs: a lingual rest and a lingual rest associated with a reciprocal arm. In addition, the indirect retention of the mandibular right canine was evaluated.

## 2 Materials & methods

A mandibular simulation model with a left extension distal edentulous area (Study Model UKT14; Kavo) was used as the definitive cast in the test. To determine the path of insertion, the cast was placed in the surveyor (Ney Surveyor; The JM Ney Company) following the technique described by Bezzon et al [27]. The proximal guiding plane and the lingual rests were prepared on the canines according to biomechanics principles and revised recommendations in the literature. The prepared cast was duplicated with silicone (Optosil/Xantopren; Heraeus Kulzer) and poured wax (ACCU Beads; Kerr) in a position corresponding to the canines. The mandibular root canine was carved to a length of 15 mm with a quadrangular section according to the method by Picosse [28]. This canine wax-up was fused with Ni-Cr alloy (Balken). The fused tooth had its “roots” brush-stroked with an even layer of Ureol 5073-A polyurethane resin (Max Epoxy; Huntsman). The elements were repositioned in the duplicate silicone mold in the position of the canines. Then, the cast was completely filled with acrylic resin (DuraLay III; Reliance), and it sat for seven days to allow the polyurethane to release any tension. After this period, the cast was placed in a pan at 20 psi (Orthoclass Clássico; Record) for 15 minutes to obtain adequate polymerization and thereby avoid the formation of air bubbles. The roots of the mandibular canines were involved, leaving the root extensions out of the cast. A pin was placed in the cast flooring to preserve the insertion axis. Thereafter, a layer (3 mm) of polyether film was applied at the distal extension of the matrix to simulate mucosal resilience [29]. The root extensions were fixed with type IV gypsum (Durone; Dentsply) to avoid any movement during the molding phases and to gain adaptation of the metal framework in the definitive cast.

**Fig. 1:**
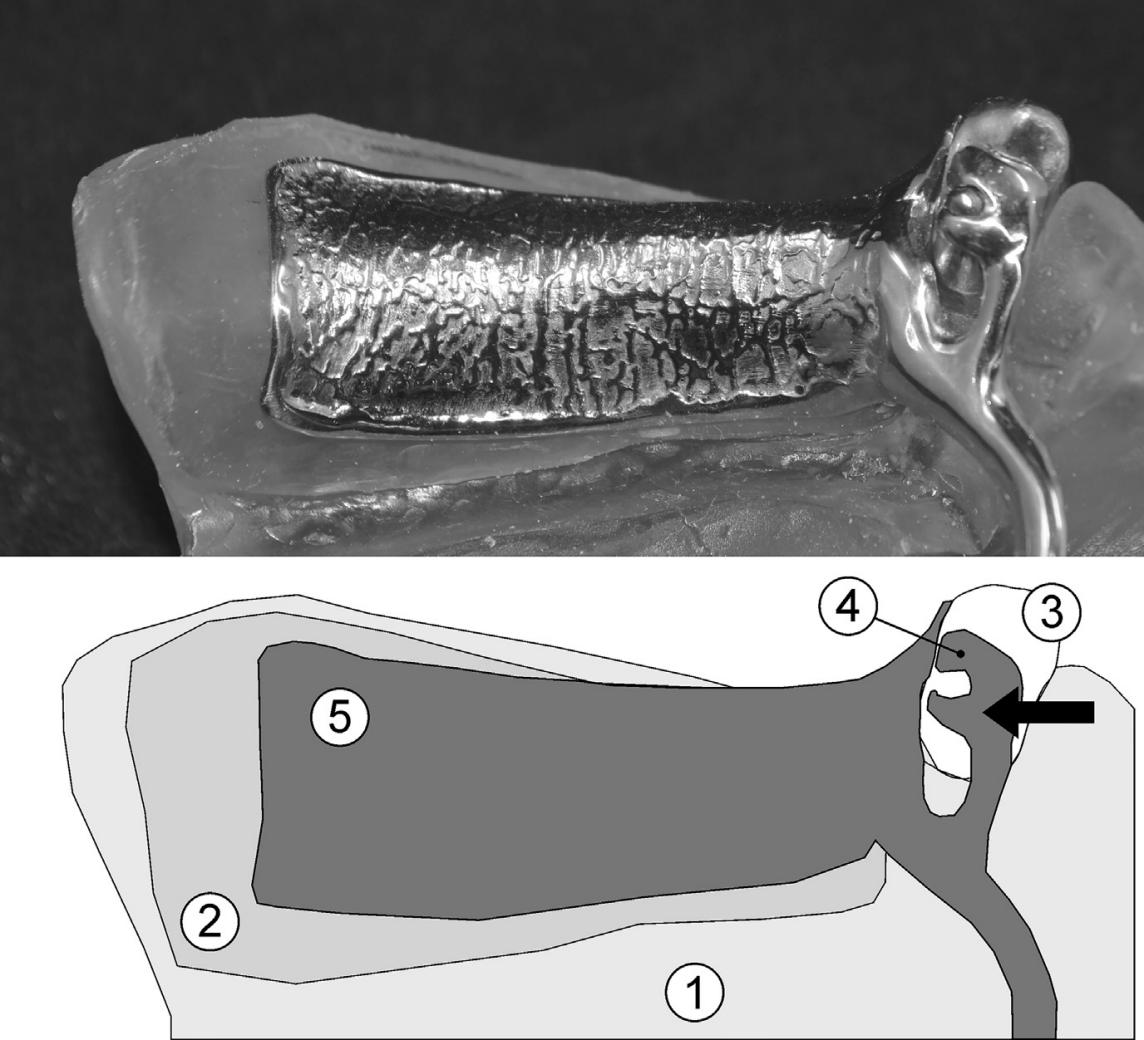
Lingual rest with reciprocal arm design. (1) Experimental cast; (2) Polyether film; (3) Canine abutment; (4) Lingual rest associated with a reciprocal arm; (5) RPDP framework.

**Fig. 2:**
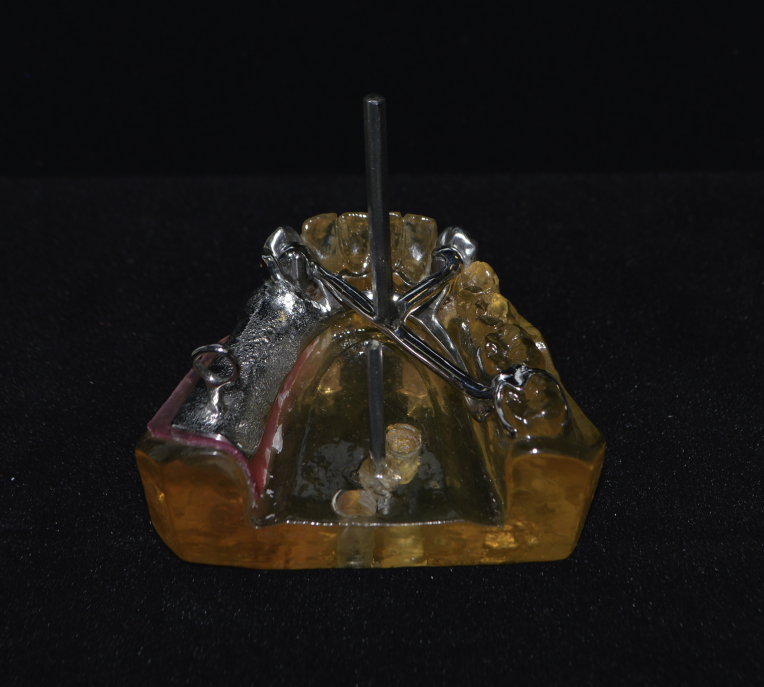
Metal framework with lingual rest after reciprocal arm removal.

A partial denture framework of Co-Cr alloy was fabricated (Biosil; Degussa Dental) with the following characteristics: a T bar clasp with a lingual rest and a reciprocal arm for the left canine (Fig. 1), a lingual rest for the right canine, and an embrasure clasp for the first and second left molars. To orient the traction through the insertion path, a steel pin obtained from a hexagonal Allen wrench type screw key (5 cm in length and 4 mm in thickness) was laser-welded with a Ni-Cr weld (Dentaurum) to the metallic framework of a transverse bar. This bar was diametrically linked to the lingual rests and was situated perpendicular to the insertion trajectory and parallel to the base of the cast. To facilitate traction of the prosthetic denture base, a pin (2 cm in length) with a ring was placed at the extremity in the position of the mandibular left second molar. Finally, to obtain the partial denture framework, the reciprocal arm was removed using a tungsten bur (Fig. 2).

The definitive cast was fixed to an aluminum base with two Allen type screws, and this base was attached to an adjustable table of the surveyor. The adjustable table was set to the guide with lateral screws and a rail, which allowed a little freedom of movement in all directions but greater movement in the mesial-distal direction, conforming to the manipulation of its screws. This system allowed the definitive cast to be fixed on the iron plate, as well as limited freedom of movement in all directions according to need, while leaving the root extensions suspended.

**Fig. 3:**
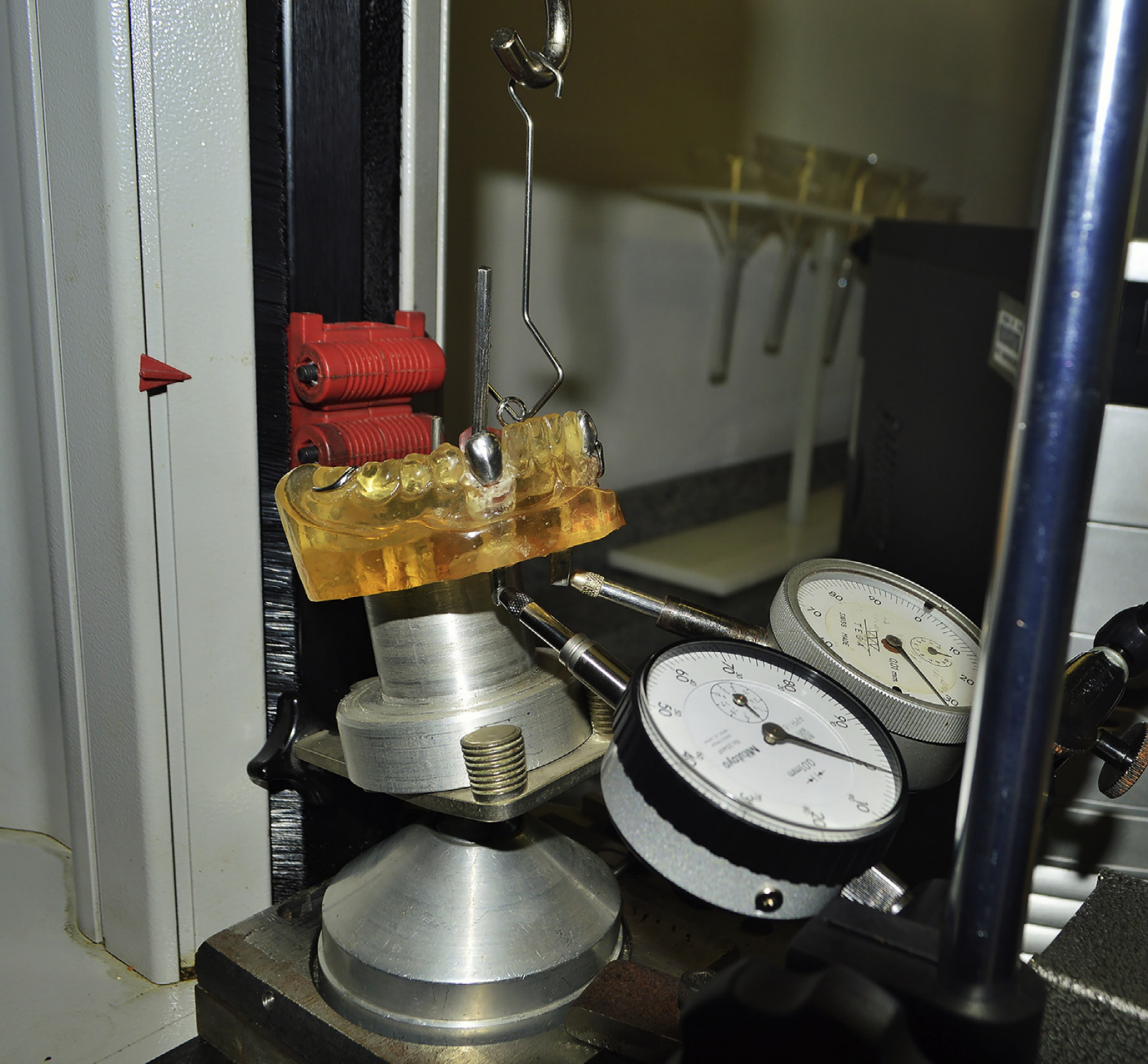
Definitive cast with experimental framework seated at the base of the VersaTest Machine. Dial gauges contact the extension root from the abutment teeth.

Each framework was subjected to tensile loading using a VersaTest traction machine (Mecmesin) with a traction speed of 5 mm per minute. We recorded the load required (N) to remove the RPDP from the definitive cast through the pin following the insertion path and the traction through the distal base denture (RPDP rotation) by means of an S-shaped metal hook. Every test was repeated twenty times for each experimental framework. The abutment movements were recorded by two dial gauges (Mitutoyo) attached to the magnetic base that were placed next to the canine root extensions (Marberg); the gauges were reset before each movement. Markings were made in millimeters, and the direction of motion was considered. If the direction of the motion was clockwise, a mark was made in the lingual and mesial directions, and if the direction of motion was counterclockwise, a mark was made in the vestibular and distal directions of the abutment movement (Fig. 3).

The statistical analysis was performed using Statistical Graphics Plus 6.0 (TIBCO Software Inc.) and Minitab 13.0 (Minitab Inc.). The data were compared using a paired t-test with 95% confidence intervals. Differences were considered significant when P < .05.

## 3 Results

In both RPDP designs, the abutment teeth were displaced in the lingual direction. The paired t-test showed significant differences regarding the amount of movement. In the RPDP design with the reciprocal arm, the movement was smaller by 0.14 ± 0.07 mm; in the design with the lingual rest, the movement was greater by 0.63 ± 0.08 mm (P < .0001) (Table 1). Furthermore, the abutment teeth were displaced in the mesial direction regardless of design, but the movement with the reciprocal arm was significantly smaller (0.26 ± 0.15 mm) than that with the lingual rest design (0.81 ± 0.06 mm; P < .0001) (Table 2).

**Table 1:** Comparative abutment movement in buccal-lingual direction (mm).

RPDP design	Mean	SD	Min	Max	*P* value
Lingual rest	0.63	0.08	0.47	0.78	<.0001^∗^
Lingual rest with reciprocal arm	0.14	0.07	0.07	0.17	

^∗^*P* < .05 (significant).

**Table 2:** Comparative abutment movement in mesial-distal direction (mm).

RPDP design	Mean	SD	Min	Max	*P* value
Lingual rest	0.81	0.06	0.6	0.89	<0.0001^∗^
Lingual rest with reciprocal arm	0.26	0.15	0.05	0.47	

^∗^*P* < 0.05 (significant).

Regarding the tensile force required to remove the RPDP framework in the insertion path, the paired t-test showed significant differences between the two designs (P < .0001). The reciprocal arm design required the greatest load to dislodge the framework (18.1 ± 4.8 N); for the lingual rest design, the required load was 35.4 ± 8.2 N (Table 3).

**Table 3:** Comparative tensile loading of frameworks (N).

RPDP design	Mean	SD	Min	Max	*P* value
Lingual rest	18.1	4.8	8	32	<0.0001^∗^
Lingual rest with reciprocal arm	35.4	8.2	20	50	

^∗^*P* < 0.05 (significant).

## 4 Discussion

The use of dial gauges to evaluate the dynamic behavior of abutment teeth has been replaced by finite element analysis, an accurate method that has proven to be an extremely powerful tool when conventional methods are not viable because of structural and material complexity [30]. Nevertheless, it is known that an RPDP has a greater expected range of motion, and a finite element analysis may be adequate for basic evaluations and analyses of devices with less expected relative movement, such as tooth- or implant-supported prostheses [31].

The data from the present study showed that the abutment tooth was displaced in the lingual and mesial directions, regardless of design, when the RPDP framework was submitted to tensile loading. Otherwise, the abutment was generally displaced in the buccal and distal directions when occlusal force was applied [16]. In clinical practice, it is important that the direction and movement of teeth be confined within the physiological limits of tooth mobility [10]. The movements of the abutment in this study did not match the parameters cited by McGivney et al. [29], who claimed that teeth have the capacity for movement of 0.25 ± 1 mm within their sockets under 4 N of force. This difference could be attributed to the presence of a 1.5-mm layer of resilient material that was used to emulate the thickness of the periodontal ligament; despite this, more thickness was added to prevent the material from tearing when subjected to traction.

With respect to movement in the buccal-lingual direction, the design with the reciprocal arm was associated with less lingual movement of the abutment tooth, demonstrating that the presence of the reciprocal arm contributes to neutralizing the harmful force exerted on the teeth by the retention arm. These results are consistent with those in other studies [23, 24, 25]. On the other hand, in the mesial-distal direction, the abutment tooth movement in both designs was in the mesial direction; this probably occurred due to the point of rotation generated by the lingual rest, which enabled activation of the T bar clasp, thus pulling the teeth in the mesial direction. This situation was also observed by Kratochvil [17].

In this study, the mean retention force in the reciprocal arm design was 36 N. Rodrigues et al. [32] previously reported a T bar clasp retentive force of 16.5 N. The high value of the retention force found in this study can be attributed to the embrasure clasp on the opposite site, the activation of the T bar clasp, and the friction provided by the reciprocal arm [23, 24, 25].

Concerning the indirect retention, the right canine moved in the mesial direction with both designs. This movement can be attributed to the rest rotation that occurs when the framework moves away from the residual ridge [33]. An indirect retainer may serve the following functions: effectively activating the direct retainer to prevent movement of a distal extension base away from tissues, tending to reduce antero-posterior tilting leverages on the principal abutments and helping to stabilize against horizontal movement of the denture [18, 19, 20].

These results are clinically important because they show canine abutment behavior in a Kennedy Class II RPDP; awareness of this behavior alerts clinicians to the importance of careful design, correct impressions for recording the residual ridge in a functional or supporting form and periodic recalls. Thus, all of these movements can be controlled within the physiological limits of the periodontal ligament. This in vitro study tried to best simulate typical oral conditions; however, there is error associated with the use of dial gauges. In addition, the resulting intrinsic factor of fatigue of the polyurethane material and involuntary factors associated with laboratory work are limitations of this study. It is important to associate these results with clinical observations that will allow more practical and less harmful planning of a distal-extension RPDP.

## 5 Conclusions

The results suggest that a lingual rest associated with a reciprocal arm may lead to decreased tooth mobility when a canine is the abutment of a Kennedy class II RPDP. The abutment tooth was displaced in the mesial and lingual direction, regardless of the type of design. Nevertheless, the lingual rest associated with a reciprocal arm significantly reduced the amplitude of these movements and enhanced RPDP retention.

## Declarations

### Author contribution statement

M. Castilla: Conceived and designed the experiments; Performed the experiments.

B. Costa: Analyzed and interpreted the data; Contributed reagents, materials, analysis tools or data.

R. Stegun: Analyzed and interpreted the data.

N. Sesma: Conceived and designed the experiments.

N. Raico: Performed the experiments; Analyzed and interpreted the data; Wrote the paper.

### Funding statement

This research did not receive any specific grant from funding agencies in the public, commercial, or not-for-profit sectors.

### Competing interest statement

The authors declare no conflict of interest.

### Additional information

No additional information is available for this paper.
